# Cabozantinib as the causative agent of high-grade fever in a patient with a background of metastatic clear cell renal cell carcinoma: a case report

**DOI:** 10.1186/s13256-020-02560-0

**Published:** 2020-11-20

**Authors:** H. M. N. Chen, M. Morris, P. M. Manders

**Affiliations:** 1 Sunshine Coast University Hospital, 6 Doherty St, Birtinya, 4575 Australia; 2 Griffith University, Sunshine Coast Health Institute, 6 Doherty St, Birtinya, 4575 Australia

**Keywords:** Cabozantinib, Renal, Fever, Case report

## Abstract

**Background:**

Fever, as an adverse event, is well documented in a wide array of drugs including multiple tyrosine kinase inhibitors however, it is not a previously well described consequence of the novel multi-targeted tyrosine kinase inhibitor, cabozantinib.

**Case presentation:**

In this paper we document the first detailed review of high-grade fevers in a 54 year old male (Caucasian) with a background of metastatic clear cell renal cell carcinoma recently commenced on cabozantinib. After detailed investigation, we exclude infection and other common causes of fever as the causative agent and further, definitively resolve the recurrent fever by ceasing cabozantinib and starting a short course of oral corticosteroids.

**Conclusions:**

We have demonstrated that cabozantinib should always be considered in the aetiology of high-grade fever in relevant patients. Further, we demonstrate that temporary cessation of cabozantinib and a course of short-term steroids can induce resolution of fever and allow for recommencement of cabozantinib safely thereafter.

## Introduction

Treatment of metastatic renal cell carcinoma (mRCC) is rapidly evolving with the introduction of new targeted therapies and ongoing investigation into combination therapies using immunotherapy backbones [1].

Vascular endothelial growth factor (VEGF) is a potent angiogenic stimulus supporting the growth of renal cell carcinoma (RCC) [2]. VEGF and associated downstream activation pathways, therefore provide a number of targets for metastatic growth inhibition [3].

Cabozantinib is an oral, small molecule, multi-targeted tyrosine kinase inhibitor (TKI) with proven activity against VEGF-Receptor (VEGFR) mediated cell cycling activation. In addition, it also inhibits both the MET and AXL tyrosine kinase pathways which have been shown to associate with poorer prognosis due to conferral of resistance to VEGFR-specific inhibition [4].

Common adverse effects of cabozantinib are similar to those typically found in earlier VEGF-specific TKI’s. These include fatigue, diarrhoea, hypertension, palmar-plantar erythrodysesthesia, anorexia and dysgeusia [4, 5].

To our knowledge, high grade fever with rigors in the absence of infection is not a well-documented sequelae of cabozantinib, although has been seen in other TKI’s, particularly for example Dabrafenib in melanoma [6]. In this report, we describe a case of refractory, high-grade fever with rigors in a patient recently commencing cabozantinib in the absence of any more plausible causative agent after detailed investigation.

## Case presentation

### Patient information, clinical findings, timeline and diagnostic assessment

A 54 year old male (Caucasian) was commenced on 4th line cabozantinib 40 mg daily in Dec 2019 for Metastatic Renal Cell Carcinoma following disease progression after 8 cycles of 3rd line pembrolizumab and denosumab which was given as per the KEYPAD trial [7]. He was originally diagnosed with de-novo metastatic RCC in April 2015 with a right sided renal lesion and multiple pulmonary metastases present on baseline imaging. He subsequently underwent a cytoreductive right nephrectomy in June 2015 which demonstrated a grade 2, 95 mm, T3aNxM1, Clear Cell RCC on histology with evidence of invasion into the proximal renal vein. First line Pazopanib was commenced in July 2015 and was continued for 11 months until disease progression. Second line Axitinib was therefore commenced July 2016 with no significant issues and was well tolerated for several years until disease progression in 2019 when he was enrolled on the aforementioned KEYPAD trial prior to commencing cabozantinib.

On this occasion he presented to our Emergency department with fevers, rigors and a concomitant widespread non-pruritic, erythematous, maculopapular rash predominantly across his torso and back, 2 weeks after commencement of cabozantinib. These symptoms were preceded by a 2-day history of worsening fatigue, myalgia and right upper quadrant abdominal pain. There was no coryzal stigmata. No sick contacts were identified despite exhaustive investigation.

Our patient was treated empirically with one dose of 1 g intravenous Cefepime in the Emergency Department before transfer to the ward. Subsequent antibiotics were withheld as no infective foci were identified and observations, excluding temperature, were within normal limits. Septic screen including urinalysis and culture, chest x-ray and repeat blood cultures remained non-contributory throughout admission. Ultrasound of the abdomen demonstrated no evidence of biliary pathology and was otherwise unremarkable. Extended respiratory viral nucleic acid detection was negative. Cabozantinib was withheld during admission.

The patient continued to spike high grade fevers up to 39.5 °C with associated rigors for 3 days, however his rash improved and rapidly subsided over 2 days with the use of topical hydrocortisone and oral antihistamines. White cell count was normal at 5.7 × 10^9^/L (reference range: 4.0–11.0 × 10^9^/L) and neutrophil count also unremarkable. C-reactive protein levels were elevated at 211 mg/L. A procalcitonin level was ordered with a result of 1.3 μg/L (reference range: 0.5–2.0 μg/L) suggesting no obvious concomitant bacterial infection. Differential diagnoses of exclusion were thus considered to be either drug related fever and/or primary tumour related fever.

### Therapeutic intervention

**Fig. 1:**
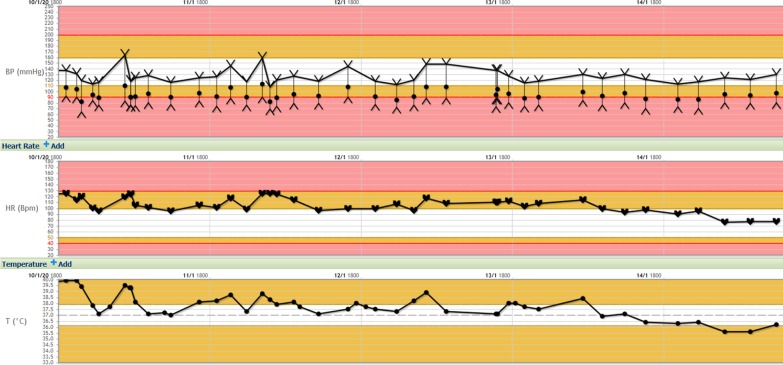
Clinical observations during the Patient’s Hospital Admission. Vital signs measured during the patient’s hospital admission including blood pressure, heart rate and temperature. The patient demonstrated high grade cyclical fevers during the admission with associated tachycardia. Improvement in these signs where seen following the administration of steroids on the 13/01/20. The last dose of cabozantinib was taken on 9/1/2020, the day prior to admission. A single dose of cefepime was administered on 10/1/2020

He was therefore commenced on 8 mg oral dexamethasone for presumed drug related fevers and remained afebrile for 24 h following administration. After quick symptomatic improvement, he was discharged on a weaning course of 4 mg daily of oral dexamethasone for a week, followed by 2 mg daily for week until he was due to be seen by his treating Oncologist (Fig. 1).

### Follow-up and outcomes

Two weeks later, he was routinely reviewed in our Outpatients Clinic following successful weaning off dexamethasone with nil further fevers or rigors. The patient was keen to recommence cabozantinib and has been re-challenged on the same dose of 40 mg daily. To date, he remains well with no evidence of fever recurrence now 35 days post discharge (Table 1).

**Table 1:** Summary of relevant investigations performed and result during patient admission

Investigation	Result
White cell count	4.3 × 10^9^/L–5.7 × 10^9^/L
Neutrophil count	3.14 × 10^9^/L–4.49 × 10^9^/L
Procalcitonin	1.3 μg/L
C-reactive protein	211 mg/L
Urine culture	No sign of infection
Blood culture (× 5)	No sign of infection
Chest X Ray	No sign of infection
Abdominal ultrasound	No sign of infection
Respiratory serology: *R.* *rickettsia*, *O. tsutsugamushi*	No sign of infection
Respiratory nucleic acid: Influenza, RSV, Parainfluenza, Adenovirus, Human Metapneumovirus,	No sign of infection
Herpesviriadae studies (CMV, EBV)	No sign of infection

*CMV* Cytomegalovirus, *EBV* Epstein Barr Virus

## Discussion

We believe this case highlights the first detailed example of cabozantinib-induced fever in the literature. Potential causes of high-grade fevers were considered, including tumour-related fevers, infection and drug-induced fevers. With regards to tumour-related fevers, RCC typically presents with fever in 20% of cases with some correlation between tumour size and extent of fever [8]. Tumours induce fever primarily through either necrosis of large tumours or through the production of pro-inflammatory cytokines [9]. Tumour-related fevers are commonly asymptomatic in up to 60% of cases and typically intermittent and low grade in nature—ranging between 38.0 and 38.9 °C [10]. In patients exhibiting tumour-related fevers, fevers are usually associated with once daily peaks and sustained baseline heart rates [10, 11]. In this case, our patient conversely demonstrated refractory high-grade fevers with concomitant tachycardia and symptomatic pyrexia and rigor. In addition, there was no large volume disease progression on recent CT restaging to suggest RCC tumour related fevers [8].

In patients with cancer, the most common source of fevers is infection, encompassing 50% or more of cases; with bacterial causes most frequently documented [9, 12]. In this case, our patient demonstrated no focal infective stigmata, and significantly, no obvious clinical deterioration in the context of persistent fever. We acknowledge that he did receive one dose of antibiotics at admission but this was quickly ceased. Whilst a single dose of antibiotics may be sufficient to control some infection’s we believe this would also most likely be associated with abrogation of the fever in concert.

Further, the procalcitonin level certainly was not consistent with a pronounced bacterial sepsis but we do note that this is a controversial marker of infection, particularly in severe blood based infections [13, 14]. Specifically, there are limitations to procalcitonin with the relatively low or slightly elevated levels detected during localized infective processes [13]. However, extensive physical examination and history was not consistent with this occurring in our patient. Taken together, we believe that, in the context of such extensive and negative septic screen, our patient’s fevers were not attributable to infection.

A hallmark of drug-induced fevers is the temporal relationship of pyrexia, on average 1 to 2 weeks following the administration of a drug [11, 15], and fevers typically subsiding with the withdrawal of the drug [15]. In our case, the patient was transitioned to 40 mg/day of cabozantinib 2 weeks after completing cycle 8 of pembroluzimab and denosumab and 15 days prior to admission. Fever associated with anti-PD-1 therapy has been widely documented in the literature [16], however our patient had been afebrile and otherwise asymptomatic throughout 8 cycles of pembroluzimab and in addition had not been administered a PD-1 inhibitor for over 2 weeks making this far less likely to be the cause of his current illness although we agree unable to completely exclude this.

To the best of our knowledge, there have been only very limited mention of fevers possibly secondary to cabozantinib. In the METEOR trial, pyrexia was a relatively uncommon adverse effect described in 3/331 participants (0.94%) on cabozantinib as opposed to 4/322 (1.24%) on Everolimus [4].

Lastly, there are some reports in the literature suggesting that immunotherapy may generate sensitisation to TKI therapy through the use of for example, Nivolumab in RCC patients [17]*.* This raises the possibility that sensitisation associated with sequencing of treatment in our patient with pembrolizumab prior to cabozantinib may have led to more profound TKI-related sequelae. We stress however that this remains controversial and has not been supported by other investigators [18].

## Conclusion

We have presented a detailed analysis of atypical fever in an extensively treated patient with a background of metastatic RCC. We believe this is the first such report in the literature. We have identified the next generation oral TKI, cabozantinib, as the most-likely primary source of these putative drug-related fevers.

### Patient perspective

The patient reported that this presentation was the first occurrence of fevers since his recent commencement of cabozantinib as a 4th line systemic option and had not experienced similar symptomatology previously. His symptoms resolved rapidly following cessation of cabozantinib and commencing corticosteroids, however he remained keen to recommence cabozantinib as soon as possible considering his advanced metastatic RCC. He has since tolerated rechallenge of his cabozantinib without recurrence of fevers.

## Data Availability

Not applicable.
